# Association of Digoxin Application Approaches With Long-Term Clinical Outcomes in Rheumatic Heart Disease Patients With Heart Failure: A Retrospective Study

**DOI:** 10.3389/fcvm.2021.711203

**Published:** 2021-09-20

**Authors:** Cheng Liu, Yanxian Lai, Tianwang Guan, Qingchun Zeng, Jingxian Pei, Shenghui Zhang, Daihong Wu, Deping Wu

**Affiliations:** ^1^Department of Cardiology, Guangzhou First People's Hospital, South China University of Technology, Guangzhou, China; ^2^Department of Cardiology, Guangzhou First People's Hospital, Guangzhou Medical University, Guangzhou, China; ^3^Department of Cardiology, Nanfang Hospital, Southern Medical University, Guangzhou, China; ^4^Department of Cardiology, The Second Affiliated Hospital of Guangzhou Medical University, Guangzhou, China; ^5^Guangzhou Center for Disease Control and Prevention, Guangzhou, China

**Keywords:** heart failure re-hospitalization, mortality, rheumatic heart disease, application approaches, digoxin, new-onset atrial fibrillation

## Abstract

**Objective:** This retrospective, case–control study was executed to assess the effects of digoxin (DGX) use approaches [continuous use of DGX (cDGX) vs. intermittent use of DGX (iDGX)] on the long-term prognosis in rheumatic heart disease (RHD) patients with heart failure (HF).

**Methods:** A total of 642 RHD patients were enrolled to this study after propensity matching. The associations of DGX application approaches with the risks of all-cause mortality, cardiovascular death (CVD), HF re-hospitalization (1-, 3-, and 5-year), and new-onset atrial fibrillation (AF) were analyzed by multivariate Cox proportional hazards or binary logistic regression models, respectively.

**Results:** cDGX was associated with increased risks of all-cause mortality (adjusted HR = 1.84, 95% CI: 1.27–2.65, *P* = 0.001) and CVD (adjusted HR = 2.23, 95% CI: 1.29–3.83, *P* = 0.004) in RHD patients with HF compared to iDGX. With exception of 1-year HF re-hospitalization risk, cDGX was associated with increased HF re-hospitalization risk of 3-year (adjusted OR = 1.53, 95% CI: 1.03–2.29, *P* = 0.037) and 5-year (adjusted OR = 1.61, 95% CI: 1.05–2.50, *P* = 0.031) as well as new-onset AF (adjusted OR = 2.06, 95% CI: 1.09–3.90, *P* = 0.027).

**Conclusion:** cDGX was significantly associated with increased risks of all-cause mortality, CVD, medium-/long-term HF re-hospitalization, and new-onset AF in RHD patients with HF.

## Introduction

Rheumatic heart disease (RHD) is a rheumatic fever–induced acquired heart disease. It remains one of the most major causes of mortality in low- and middle-income countries. Heart failure (HF) is an independent predictor of death in RHD patients (1). Congestion is one of the classic clinical features of decompensated HF, manifesting the characteristics of extracellular fluid accumulation (2). Digoxin (DGX), as an ancient drug for HF treatment, improves cardiac output and relieves congestion owing to its positive inotropic effect. However, the evidence of DGX on the HF prognosis mainly comes from non-valvular HF rather than valvular HF and has been controversial since its use for congestive HF treatment. Early randomized controlled trials (RCTs) with small sample sizes showed that DGX improved the symptoms of HF patients (3). The Digitalis Investigation Group (DIG) study, as the only large-scale RCT with DGX for HF therapy, reported that DGX did not reduce all-cause mortality in patients with HF during 37-month follow-up (4). Based on the database of the DIG trial, a cluster analysis found that DGX did not reduce HF hospitalization risk in specific subparticipants who were female and had hypertension or left ventricular ejection fraction (LVEF) >45% and even increased mortality (5). The Valsartan Heart Failure Trial (Val-HeFT) study showed that continuous DGX treatment was related to elevated risk of all-cause death, first morbid event, and HF hospitalization (6). A 2-year retrospective analysis by Freeman et al. (7) also found that continuous DGX treatment in HF patients was independently related to a higher mortality. Recent *post-hoc* randomization analysis of the DIG trial further confirmed that the mortality was significantly increased in subparticipants pre-treated with DGX (8). Meanwhile, another meta-analysis also showed that DGX was related to elevated risk of all-cause death in HF patients (9). These findings suggested that HF patients cannot benefit from DGX use (especially continuous DGX use) and DGX use can even be harmful.

One overlooked reason of this phenomenon is possibly related to the administration strategies of DGX, including continuous use of DGX (cDGX) and intermittent use of DGX (iDGX). According to the congestion status of HF patients, cDGX is defined as continuous use of DGX even after congestion is eliminated. iDGX is defined as adopting de-escalating therapy after the congestion is eliminated, which the DGX dose is gradually reduced and eventually discontinued. Observational and RCT studies showed an opposite association between cDGX and mortality as mentioned above. On the other hand, the Prospective Randomized Study of Ventricular Failure and the Efficacy of Digoxin (PROVED) (10) and Randomized Assessment of Digoxin on Inhibitors of Angiotensin-Converting Enzyme (RADIANCE) (11) studies were designed to assess the effect of DGX withdrawal on patients with stable systolic HF [New York Heart Association (NYHA) II–III and LVEF ≤ 35%]; they found that DGX withdrawal did not improve the life quality of patients with HF. Because the two studies were not designed to evaluate the “hard endpoints” related to DGX use and also based on the short-term follow-up, small sample size, and insufficient dose of renin–angiotensin system inhibitors (RSIs) at the baseline level, it still cannot be confirmed whether iDGX might be helpful for improving survival in HF patients, but at least it did not increase mortality. Simultaneously, some studies tried to explore the effect of reducing the dosage of DGX on prognosis of patients with HF. Ahmed et al. (12) reported that a low dose (125 μg po qod) of DGX was related to lower risk of mortality and hospitalization under condition of lower serum DGX concentration (SDC). In dialysis patients with HF receiving lower-dose DGX treatment (62.5 μg po qod), patients with iDGX had fewer DGX-related poisoning symptoms and were safer compared to those with cDGX (13). These findings suggested that the DGX use in HF management may be dynamic and change with congestion status. Based on DGX de-escalation therapy, iDGX may be a potential preferred approach for HF management.

However, the influence of this strategy on the clinical prognosis in HF patients is still unclear, especially in valvular HF. Current guidelines still recommend DGX for the treatment of RHD patients with congestive HF, but there is no evidence that it helps to improve long-term prognostic progress in RHD patients with HF. In fact, prescription of DGX is still subjective and unproven to a large extent in RHD patients with congestive HF. The Rheumatic Heart Disease Global Registry (REMEDY) study (14), as the only clinical trial to evaluate the effect of DGX use on clinical endpoints among RHD patients, showed that DGX use significantly increased the mortality of the overall population as well as recurrent HF *via* 2-year follow-up. However, this retrospective study did not distinguish the effect the administration approaches of DGX on prognosis in RHD patients with HF according to the congestive status. Whether the long-term prognosis of RHD patients with HF can be improved by the de-escalation therapy of DGX, it still lacks conclusive evidence from a large well-designed RCT with “hard” clinical endpoints. Hence, the aim of this study is to access the associations between DGX use approaches (cDGX vs. iDGX) and long-term clinical prognosis in RHD patients with HF.

## Methods

### Study Subjects

A retrospective propensity score–matched case–control study from January 1, 1999, to December 31, 2020, was conducted at South China University of Technology. A total of 734 RHD patients were recruited into the study; 219 patients died during follow-up. Clinical information was collected from the four information sources, including interviews of patients and their families, review of medical records, contact with treating physicians, and the Center for Disease Control and Prevention. RHD was identified referring to the recommendations of the World Heart Federation. HF was diagnosed according to the Framingham Heart Study's criteria and American College of Cardiology (ACC)/American Heart Association (AHA) guidelines. Congestion in HF was evaluated referring to European Society of Cardiology (ESC) guidelines (2). According to the application approaches of DGX in RHD patients with HF, RHD patients were divided into two groups: cDGX (*N* = 386) and iDGX (*N* = 348). The cDGX group of participants receives continued DGX therapy regardless of whether the congestion is eliminated. The iDGX group of participants only receives DGX therapy when congestion occurs. In particular, surgical intervention was defined as valve replacement or repair surgery *via* surgical or percutaneous intervention for any affected valve(s) with tissue or mechanical prosthesis referring to recommendations. Coexisting medical conditions, including AF, hypertension (HT), coronary heart disease (CHD), stroke, and type 2 diabetes (T2D), were also diagnosed referring to relevant recommendations. Blood sample analyses were executed by standard lab protocols. Echocardiography was carried out according to our previous protocol (15). The date of entry into the study was the RHD first diagnosis date. Survival duration was calculated from the date of initial diagnosis of RHD to the date of mortality or to the date of last follow-up (December 31, 2020).

### Primary and Secondary Outcomes

All-cause death and cardiovascular death (CVD) were the primary outcomes. The all-cause death was defined as death due to any causes. The CVD was defined as death due to any cardio-/cerebrovascular diseases, such as CHD, HF, AF, stroke, and/or other cardio-/cerebrovascular causes. HF re-hospitalization and new-onset AF were the secondary outcomes. After first discharge for HF, HF re-hospitalization was defined as hospital re-hospitalization with HF treatment. The new-onset AF was defined as AF that first occurred during follow-up period.

### Propensity Score Matching and Statistical Analysis

The 1:1 propensity score matching (PSM) and statistical analyses were executed by SPSS version 24 (SPSS, Chicago, IL). The 1:1 PSM was executed referring to our previous methods (16) by baseline characteristics at enrollment such as age at diagnosis, age of surgery, disease duration, follow-up time, levels of systolic/diastolic blood pressure (SBP/DBP) and heart rate (Hr), surgical intervention, cardiac valve damage, NYHA, medical condition, combined medication, blood test index, and echocardiographic index. The caliper width was 0.1 for PSM. Categorical and continuous variables were shown as numbers (percentages) and mean ± standard deviation (SD), respectively. Significant differences for categorical variables and continuous variables were determined by χ^2^ test and independent-sample *t*-test, respectively. The multivariate Cox proportional hazards regression model was carried out to access the association [hazard ratio (HR) and 95% confidence interval (CI)] of different DGX use methods with all-cause death and CVD, adjusting by baseline characteristics. Binary logistic regression modeling was carried out to evaluate the association [odds ratio (OR) and 95% CI] of different DGX use methods with 1-, 3-, and 5-year HF readmission as well as new-onset AF. A *P*-value of 0.05 or less was statistically significant (two-tailed).

## Results

### Characteristics of Study Subjects

Before PSM, a total of 734 patients with RHD were enrolled to this study, the median follow-up time was 5.8-years among those participants, and there were obvious differences between cDGX and iDGX in RHD patients on levels of SBP (*P* = 0.025), DBP (*P* = 0.004), Hr (*P* = 0.042), the constituent ratio of mitral stenosis (MS, *P* = 0.044), surgical intervention for valve repair (*P* = 0.014), NYHA (*P* < 0.001), coexisting disorder [e.g., HT (*P* = 0.019) and AF (*P* = 0.011)], drug use [e.g., diuretics (*P* < 0.001), RSIs (*P* = 0.010), mineralocorticoid receptor antagonist (MRA, *P* < 0.001), and calcium channel blockers (CCBs, *P* = 0.037)], and echocardiographic index for right ventricular end-diastolic diameter (RVD, *P* = 0.001), left atrial end-systolic diameter (LAD, *P* = 0.001), and LVEF (*P* = 0.009), as shown in Tables 1, 2. After 1:1 PSM, a total of 642 patients with RHD were finally entered to the study with median follow-up of 5.9-years, and there was still significant difference on NYHA (*P* = 0.001) between the two groups.

**Table 1:** Baseline clinical characteristics of study subjects.

		**Before PSM**			**After PSM**		
		**iDGX (*N =* 348)**	**cDGX (*N =* 386)**	***P*** **-value**	**iDGX (*N =* 321)**	**cDGX (*N =* 321)**	***P*** **-value**
Male: female		105:243	126:260	0.472	95:226	99:222	0.731
Age at diagnosis (years)		47.4 ± 16.2	45.6 ± 15.5	0.117	47.2 ± 16.3	45.7 ± 15.4	0.206
Age of surgery (years)		47.1 ± 11.1	45.6 ± 15.5	0.904	47.4 ± 11.0	47.7 ± 11.5	0.852
Disease duration (years, *N*/%)	Duration <1	68 (19.5)	60 (15.5)	0.176	55 (17.1)	53 (16.5)	0.402
	1 ≤ Duration < 5	86 (24.7)	84 (21.8)		78 (24.3)	68 (21.2)	
	5 ≤ Duration < 10	52 (14.9)	55 (14.2)		49 (15.3)	42 (13.1)	
	10 ≤ Duration < 15	62 (17.8)	69 (17.9)		60 (18.7)	57 (17.8)	
	Duration ≥ 15	80 (23.0)	118 (30.6)		79 (24.6)	101 (31.5)	
Follow-up (years)		5.9 (3.5, 9.3)	5.6 (3.2, 9.7)	0.701	6.1 (3.6, 9.5)	5.7 (3.3, 9.8)	0.598
Average daily dose of DGX (μg)		125 (125, 125)	125 (125, 125)	0.423	125 (125, 125)	125 (125, 125)	0.872
Cumulative use time of DGX (years)		4.0 (1.8, 6.7)	5.7 (2.7, 9.0)	<0.001	3.1 (1.4, 6.5)	5.7 (3.3, 9.7)	<0.001
Smoking (*N*/%)		35 (10.1)	47 (12.2)	0.363	29 (9.0)	38 (11.8)	0.245
Drinking (*N*/%)		39 (11.2)	48 (12.4)	0.607	33 (10.3)	39 (12.1)	0.453
SBP at initial diagnosis (mmHg)		**119.9 ± 10.0**	**121.6 ± 11.1**	**0.025**	119.9 ± 10.2	121.5 ± 11.4	0.070
DBP at initial diagnosis (mmHg)		**72.6 ± 10.7**	**74.9 ± 10.8**	**0.004**	72.5 ± 10.8	73.2 ± 8.8	0.364
HR at initial diagnosis (bpm)		**82 ± 18**	**85 ± 17**	**0.042**	82 ± 18	84 ± 16	0.175
**Cardiac valve damage (*N*/%)**							
(AI) Valve damage (stenosis or regurgitation) types (single: combined)		165:183	170:216	0.360	163:158	151:170	0.343
(A-II) Types of single valve damage	MV	153 (92.7)	158 (92.9)	0.090	151 (92.6)	140 (92.7)	0.109
	AV	8 (4.8)	12 (7.1)		8 (4.9)	11 (7.3)	
	TV	4 (2.4)	0 (0.0)		4 (2.5)	0 (0.0)	
(A-III) Types of combined valve damage	MV+AV	99 (54.1)	105 (48.6)	0.506	78 (49.4)	81 (47.6)	0.919
	MV+TV	49 (26.8)	68 (31.5)		46 (29.1)	53 (31.2)	
	MV+AV+TV	35 (19.1)	43 (19.9)		34 (21.5)	36 (21.2)	
(B-I) MS		245 (70.4)	297 (76.9)	0.044	228 (71.0)	245 (76.3)	0.128
(B-II) MS degree	Mild	135 (55.1)	160 (53.9)	0.813	125 (54.8)	135 (55.1)	0.669
	Moderate	69 (28.2)	81 (27.3)		67 (29.4)	65 (26.5)	
	Severe	41 (16.7)	56 (18.9)		36 (15.8)	45 (18.4)	
(C-I) MR		210 (60.3)	208 (53.9)	0.078	198 (61.7)	178 (55.5)	0.109
(C-II) MR degree	Mild	75 (35.7)	80 (38.5)	0.495	69 (34.8)	67 (37.6)	0.582
	Moderate	58 (27.6)	47 (22.6)		56 (28.3)	42 (23.6)	
	Severe	77 (36.7)	81 (38.9)		73 (36.9)	69 (38.8)	
(D-I) AS		84 (24.1)	94 (24.4)	0.946	74 (23.1)	78 (24.3)	0.710
(D-II) AS degree	Mild	50 (59.5)	50 (53.2)	0.445	43 (58.1)	41 (52.6)	0.591
	Moderate	25 (29.8)	28 (29.8)		22 (29.7)	23 (29.5)	
	Severe	9 (10.7)	16 (17.0)		9 (12.2)	14 (17.9)	
(E-I) AR		125 (35.9)	136 (35.2)	0.846	108 (33.6)	109 (34.0)	0.934
(E-II) AR degree	Mild	60 (48.0)	62 (45.6)	0.437	53 (49.1)	48 (44.0)	0.374
	Moderate	48 (38.4)	61 (44.9)		40 (37.0)	50 (45.9)	
	Severe	17 (13.6)	13 (9.6)		15 (13.9)	11 (10.1)	
(F-I) TR		88 (25.3)	111 (28.8)	0.291	84 (26.2)	89 (27.7)	0.656
(F-II) TR degree	Mild	29 (33.0)	39 (35.1)	0.089	27 (32.1)	31 (34.8)	0.137
	Moderate	35 (39.8)	29 (26.1)		34 (40.5)	24 (27.0)	
	Severe	24 (27.3)	43 (38.7)		23 (27.4)	34 (38.2)	
(G-I) PAH		241 (69.3)	285 (73.8)	0.169	227 (70.7)	239 (74.5)	0.228
(G-II) PAH degree	Mild	141 (58.5)	154 (54.0)	0.161	130 (57.3)	134 (56.3)	0.657
	Moderate	72 (29.9)	81 (28.4)		70 (30.8)	69 (29.0)	
	Severe	28 (11.6)	50 (17.5)		27 (11.9)	35 (14.7)	
**Surgical intervention (*N*/%)**							
(A) Valve replacement (tissue or mechanical prosthesis)	MV	109 (65.7)	145 (70.4)	0.265	107 (70.4)	120 (71.4)	0.450
	AV	5 (3.0)	12 (5.8)		4 (2.6)	10 (6.0)	
	MV+AV	49 (29.5)	47 (22.8)		39 (25.7)	36 (21.4)	
	MV+TV	3 (1.8)	2 (1.0)		2 (1.3)	2 (1.2)	
(B) Valve repair	MV	**18 (69.2)**	**12 (41.4)**	**0.014**	18 (69.2)	8 (47.1)	0.094
	TV	**5 (19.2)**	**17 (58.6)**		5 (19.2)	9 (52.9)	
	AV	**2 (7.7)**	**0 (0.0)**		2 (7.7)	0 (0.0)	
	MV+TV	**1 (3.8)**	**0 (0.0)**		1 (3.8)	0 (0.0)	
**NYHA (*N*/%)**	I	**45 (12.9)**	**49 (12.7)**	** <0.001**	**41 (12.8)**	**49 (15.3)**	**0.001**
	II	**133 (38.2)**	**102 (26.4)**		**126 (39.3)**	**87 (27.1)**	
	III	**138 (39.7)**	**165 (42.7)**		**124 (38.6)**	**128 (39.9)**	
	IV	**30 (9.2)**	**70 (18.1)**		**30 (9.3)**	**57 (17.8)**	
**Medical condition (*N*/%)**							
HT		**102 (29.3)**	**84 (21.8)**	**0.019**	93 (29.0)	78 (24.3)	0.181
CHD		20 (5.7)	32 (8.3)	0.180	17 (5.3)	26 (8.1)	0.155
T2D		42 (12.1)	35 (9.1)	0.185	38 (11.8)	30 (9.3)	0.305
AF		**122 (35.1)**	**171 (44.2)**	**0.011**	119 (37.1)	134 (41.7)	0.226
Stroke		40 (11.5)	41 (10.6)	0.706	39 (12.1)	38 (11.8)	0.903
**Combined medication (*N*/%)**							
Antiplatelet drugs		60 (17.2)	60 (15.5)	0.535	56 (17.4)	52 (16.2)	0.673
Warfarin		218 (62.6)	216 (56.0)	0.066	202 (62.9)	182 (56.7)	0.107
Diuretics		**182 (52.3)**	**280 (66.4)**	** <0.001**	181 (56.4)	204 (63.6)	0.064
Nitrates		42 (12.1)	56 (14.5)	0.332	42 (13.1)	48 (15.0)	0.495
RSIs		**171 (49.1)**	**153 (39.6)**	**0.010**	154 (48.0)	146 (45.5)	0.527
BBs		127 (36.5)	120 (31.1)	0.122	118 (36.8)	107 (33.3)	0.363
MRA		**176 (50.6)**	**249 (64.5)**	** <0.001**	172 (53.6)	196 (61.1)	0.055
CCBs		**42 (12.1)**	**29 (7.5)**	**0.037**	38 (11.8)	28 (8.7)	0.194
Statins		40 (11.5)	41 (10.6)	0.706	37 (11.5)	36 (11.2)	0.901

*The bold values mean P value < 0.05*.

**Table 2:** Baseline laboratory and echocardiography profile of study subjects.

		**Before PSM**			**After PSM**		
		**iDGX (*N =* 348)**	**cDGX (*N =* 386)**	***P*** **-value**	**iDGX (*N =* 321)**	**cDGX (*N =* 321)**	***P*** **-value**
**Blood biochemical index**							
WBC (× 10^9^/L)		7.46 ± 2.93	7.88 ± 3.36	0.068	7.42 ± 2.98	7.88 ± 3.48	0.076
HGB (g/L)		121.6 ± 18.8	121.9 ± 18.9	0.812	121.9 ± 18.9	121.7 ± 18.8	0.861
PLT (× 10^9^/L)		190.7 ± 72.0	186.2 ± 71.4	0.398	190.6 ± 71.9	191.1 ± 72.6	0.941
FBG (mmol/L)		5.84 ± 2.35	5.61 ± 2.01	0.159	5.67 ± 1.99	5.52 ± 1.92	0.361
ALT (U/L)		22.0 ± 18.6	23.6 ± 29.6	0.406	21.5 ± 16.4	22.6 ± 20.7	0.419
AST (U/L)		31.3 ± 23.2	33.2 ± 24.5	0.309	31.2 ± 22.8	33.1 ± 24.2	0.343
Cr (μmol/L)		86.8 ± 33.0	87.2 ± 36.4	0.878	87.1 ± 33.5	87.4 ± 36.5	0.905
CRP (mg/L)		15.4 ± 26.3	14.6 ± 27.8	0.723	15.5 ± 26.5	15.9 ± 30.0	0.882
ASO (U/ml)		65.3 ± 85.7	47.7 ± 69.7	0.088	66.2 ± 85.4	45.9 ± 58.7	0.053
RF (U/ml)		14.6 ± 31.7	9.5 ± 16.3	0.127	14.9 ± 32.5.8	9.0 ± 17.2	0.112
ESR (mm/h)		25.2 ± 24.4	21.5 ± 17.8	0.147	25.6 ± 24.8	21.8 ± 18.4	0.169
BNP (pg/ml)		1,031.5 ± 2,615.4	1,210.1 ± 3,130.2	0.410	1,031.6 ± 2,655.1	1,148.6 ± 3,165.1	0.615
TRIG (mmol/L)		1.23 ± 0.83	1.23 ± 1.17	0.964	1.19 ± 0.713	1.27 ± 1.26	0.322
TC (mmol/L)		4.25 ± 1.18	4.36 ± 1.30	0.246	4.27 ± 1.18	4.41 ± 1.30	0.143
LDL-C (mmol/L)		2.43 ± 0.90	2.45 ± 0.95	0.879	2.45 ± 0.91	2.48 ± 0.92	0.669
HDL-C (mmol/L)		1.12 ± 0.35	1.16 ± 0.79	0.358	1.14 ± 0.33	1.12 ± 0.34	0.490
Na^+^ (mmol/L)		139.5 ± 4.7	139.3 ± 4.1	0.508	139.6 ± 4.7	139.4 ± 4.2	0.657
K^+^ (mmol/L)		3.95 ± 0.44	3.99 ± 0.47	0.243	3.95 ± 0.44	3.96 ± 0.47	0.845
INR ratio	INR <2.0	35 (10.8)	59 (16.5)	0.055	34 (11.3)	50 (16.8)	0.096
	2.0 ≤ INR <3.0	285 (87.7)	289 (81.0)		262 (87.0)	240 (80.5)	
	INR ≥ 3.0	5 (1.5)	9 (2.5)		5 (1.7)	8 (2.7)	
**Echocardiography**	LVD (cm)	4.58 ± 0.68	4.66 ± 0.79	0.116	4.58 ± 0.68	4.63 ± 0.76	0.369
	LAD (cm)	**4.55 ± 0.80**	**4.75 ± 0.82**	**0.001**	4.57 ± 0.80	4.71 ± 0.64	0.502
	RVD (cm)	**2.24 ± 0.81**	**2.05 ± 0.74**	**0.001**	2.09 ± 0.56	2.02 ± 0.71	0.180
	RAD (cm)	3.59 ± 0.64	3.67 ± 0.76	0.133	3.59 ± 0.65	3.64 ± 0.72	0.436
	LVEF (%)	**57.4 ± 8.5**	**55.6 ± 9.1**	**0.009**	57.3 ± 8.7	56.2 ± 8.9	0.111

*The bold values mean P value < 0.05*.

### Association of DGX Application Methods With All-Cause Death and CVD in RHD Patients

After PSM, cDGX was associated with elevated risks of all-cause death (unadjusted HR = 2.49, 95% CI: 1.85–3.34, *P* < 0.001) and CVD (unadjusted HR = 3.51, 95% CI: 2.23–5.30, *P* < 0.001) in RHD patients with HF compared to those with iDGX. Adjusting for confounding covariates, cDGX was still correlated with increased risks of all-cause death (model 1: adjusted HR = 1.86, 95% CI: 1.31–2.65, *P* = 0.001; model 2: adjusted HR = 1.81, 95% CI: 1.26–2.60, *P* = 0.001; model 3: adjusted HR = 1.84, 95% CI: 1.27–2.65, *P* = 0.001) and CVD (model 1: adjusted HR = 2.46, 95% CI: 1.47–4.12, *P* = 0.001; model 2: adjusted HR = 2.33, 95% CI: 1.36–3.99, *P* = 0.002; model 3: adjusted HR = 2.23, 95% CI: 1.29–3.83, *P* = 0.004) in RHD patients with HF, as shown in Table 3.

**Table 3:** Association of DGX application methods with all-cause death and CVD in RHD patients.

**Outcomes**		**Group**	**Before PSM**			**After PSM**		
			**Events (*N*/%)**	**HR (95% CI)**	***P*** **-value**	**Events (*N*/%)**	**HR (95% CI)**	***P*** **-value**
All-cause mortality	Unadjusted	iDGX	68 (19.5)	Ref.		66 (20.6)	Ref.	
		cDGX	151 (39.1)	2.48 (1.87–3.31)	<0.001	130 (40.5)	2.49 (1.85–3.34)	<0.001
	Model 1^a^	iDGX	68 (19.5)	Ref.		66 (20.6)	Ref.	
		cDGX	151 (39.1)	1.88 (1.34–2.65)	<0.001	130 (40.5)	1.86 (1.31–2.65)	0.001
	Model 2^b^	iDGX	68 (19.5)	Ref.		66 (20.6)	Ref.	
		cDGX	151 (39.1)	1.73 (1.22–2.59)	0.002	130 (40.5)	1.81 (1.26–2.60)	0.001
	Model 3^c^	iDGX	68 (19.5)	Ref.		66 (20.6)	**Ref**.	
		cDGX	151 (39.1)	1.82 (1.28–2.59)	0.001	130 (40.5)	**1.84 (1.27–2.65)**	**0.001**
CVD	Unadjusted	iDGX	32 (9.2)	Ref.		31 (9.7)	Ref.	
		cDGX	105 (27.2)	3.65 (2.46–5.42)	<0.001	87 (27.1)	3.51 (2.33–5.30)	<0.001
	Model 1^a^	iDGX	32 (9.2)	Ref.		31 (9.7)	Ref.	
		cDGX	105 (27.2)	2.50 (1.54–4.04)	<0.001	87 (27.1)	2.46 (1.47–4.12)	0.001
	Model 2^b^	iDGX	32 (9.2)	Ref.		31 (9.7)	Ref.	
		cDGX	105 (27.2)	2.35 (1.42–3.89)	0.001	87 (27.1)	2.33 (1.36–3.99)	0.002
	Model 3^c^	iDGX	32 (9.2)	Ref.		31 (9.7)	**Ref**.	
		cDGX	105 (27.2)	2.24 (1.35–3.73)	0.002	87 (27.1)	**2.23 (1.29–3.83)**	**0.004**

a *Model 1: adjusting for baseline adjustment covariates, including age, gender, smoking, drinking, disease duration, NYHA, cardiac valve damage, surgical intervention, medical condition (HT, CHD, T2D, AF at enrolment, and stroke), blood biochemical index (WBC, HGB, PLT, FBG, ALT, AST, Cr, ASO, RF, ESR, BNP, TRIG, TC, LDL-C, HDL-C, serum sodium, and serum potassium), and echocardiography (LAD, LVD, RVD, RAD, and LVEF).*

b *Model 2: It was the same as model 1 and also included new-onset AF and combined medication (antiplatelet drugs, warfarin, nitrates, RSIs, BBs, MRAs, CCBs, and statins).*

c *Model 3: It was the same as model 2 and also included SDC.*

*The bold values mean P value <0.05*.

### Association of DGX Application Methods With HF Re-hospitalization in RHD Patients

After PSM, cDGX was associated with elevated HF re-hospitalization risks of 1-year (unadjusted OR = 1.54, 95% CI: 1.05–2.26, *P* = 0.026), 3-year (unadjusted OR = 1.69, 95% CI: 1.20–2.38, *P* = 0.003), and 5-year (unadjusted OR = 2.08, 95% CI: 1.44–3.02, *P* < 0.001) in RHD patients with HF compared to those with iDGX. Adjusting for confounding covariates, cDGX was still correlated with increased HF readmission risk of 3-year (model 1: adjusted OR = 1.55, 95% CI: 1.08–2.22, *P* = 0.018; model 2: adjusted OR = 1.62, 95% CI: 1.08–2.42, *P* = 0.020; model 3: adjusted OR = 1.53, 95% CI: 1.03–2.29, *P* = 0.037) and 5-year (model 1: adjusted OR = 1.98, 95% CI: 1.34–2.94, *P* = 0.001; model 2: adjusted OR = 1.75, 95% CI: 1.14–2.68, *P* = 0.011; model 3: adjusted OR = 1.61, 95% CI: 1.05–2.50, *P* = 0.031), as shown in Table 4.

**Table 4:** Association of DGX application methods with HF re-hospitalization in RHD patients.

**HF re-hospitalization (*N*/%)**		**Before PSM**				**After PSM**			
		**iDGX (Ref.)**	**cDGX**	**OR (95% CI)**	***P*** **-value**	**iDGX (Ref.)**	**cDGX**	**OR (5% CI)**	***P*** **-value**
Unadjusted	1-year	62 (18.1)	102 (28.1)	1.77 (1.23–2.52)	0.002	59 (42.8)	79 (57.2)	1.54 (1.05–2.26)	0.026
	3-year	108 (35.9)	165 (52.4)	1.97 (1.42–2.72)	<0.001	101 (44.1)	128 (55.9)	1.69 (1.20–2.38)	0.003
	5-year	138 (51.7)	206 (70.3)	2.21 (1.56–3.13)	<0.001	130 (43.9)	166 (56.1)	2.08 (1.44–3.02)	<0.001
Model 1^a^	1-year	62 (18.1)	102 (28.1)	1.50 (1.03–2.18)	0.036	59 (42.8)	79 (57.2)	1.39 (0.93–2.08)	0.106
	3-year	108 (35.9)	165 (52.4)	1.75 (1.24–2.45)	0.001	101 (44.1)	128 (55.9)	1.55 (1.08–2.22)	0.018
	5-year	138 (51.7)	206 (70.3)	2.01 (1.40–2.90)	<0.001	130 (43.9)	166 (56.1)	1.98 (1.34–2.94)	0.001
Model 2^b^	1-year	62 (18.1)	102 (28.1)	1.31 (0.87–1.97)	0.191	59 (42.8)	79 (57.2)	1.25 (0.81–1.93)	0.305
	3-year	108 (35.9)	165 (52.4)	1.52 (1.05–2.21)	0.027	101 (44.1)	128 (55.9)	1.62 (1.08–2.42)	0.020
	5-year	138 (51.7)	206 (70.3)	1.67 (1.12–2.54)	0.012	130 (43.9)	166 (56.1)	1.75 (1.14–2.68)	0.011
Model 3^c^	1-year	62 (18.1)	102 (28.1)	1.29 (0.85–1.94)	0.230	59 (42.8)	79 (57.2)	1.22 (0.79–1.88)	0.366
	3-year	108 (35.9)	165 (52.4)	1.51 (1.04–2.19)	0.030	101 (44.1)	128 (55.9)	**1.53 (1.03–2.29)**	**0.037**
	5-year	138 (51.7)	206 (70.3)	1.68 (1.11–2.52)	0.013	130 (43.9)	166 (56.1)	**1.61 (1.05–2.50)**	**0.031**

a *Model 1: adjusting for baseline adjustment covariates, including age, gender, smoking, drinking, disease duration, NYHA, cardiac valve damage, surgical intervention, medical condition (HT, CHD, T2D, AF at enrolment, and stroke), blood biochemical index (WBC, HGB, PLT, FBG, ALT, AST, Cr, ASO, RF, ESR, BNP, TRIG, TC, LDL-C, HDL-C, serum sodium, and serum potassium), and echocardiography (LAD, LVD, RVD, RAD, and LVEF).*

b *Model 2: It was the same as model 1 and also included new-onset AF and combined medication (antiplatelet drugs, warfarin, nitrates, RSIs, BBs, MRAs, CCBs, and statins).*

c *Model 3: It was the same as model 2 and also included SDC.*

*The bold values mean P value < 0.05*.

### Association of DGX Application Methods With New-Onset AF in RHD Patients

After PSM, cDGX was associated with elevated new-onset AF risks (unadjusted OR = 1.80, 95% CI: 1.20–2.69, *P* = 0.004) compared to those with iDGX. Adjusting for confounding covariates, cDGX was still correlated with elevated new-onset AF risk (model 1: adjusted OR = 1.96, 95% CI: 1.07–3.58, *P* = 0.028; model 2: adjusted OR = 1.94, 95% CI: 1.05–3.57, *P* = 0.034; model 3: adjusted OR = 2.06, 95% CI: 1.09–3.90, *P* = 0.027), as shown in Table 5.

**Table 5:** Association of DGX application methods with new-onset AF in RHD patients.

**New-onset AF**		**Before PSM**			**After PSM**		
		**Events (*N*/%)**	**OR (95% CI)**	***P*** **-value**	**Events (*N*/%)**	**OR (95% CI)**	***P*** **-value**
Unadjusted model	iDGXUD	91 (40.3)	Ref.		84 (41.6)	Ref.	
	cDGX	123 (57.2)	1.98 (1.36–2.90)	<0.001	105 (56.1)	1.80 (1.20–2.69)	0.004
Model 1^a^	iDGXUD	91 (40.3)	Ref.		84 (41.6)	Ref.	
	cDGX	123 (57.2)	1.73 (1.12–2.68)	0.014	105 (56.1)	1.96 (1.07–3.58)	0.028
Model 2^b^	iDGXUD	91 (40.3)	Ref.		84 (41.6)	Ref.	
	cDGX	123 (57.2)	1.58 (1.03–2.44)	0.038	105 (56.1)	1.94 (1.05–3.57)	0.034
Model 3^c^	iDGXUD	91 (40.3)	Ref.		84 (41.6)	Ref.	
	cDGX	123 (57.2)	1.61 (1.03–2.52)	0.036	105 (56.1)	**2.06 (1.09–3.90)**	**0.027**

a *Model 1: adjusting for baseline adjustment covariates, including age, gender, smoking, drinking, disease duration, NYHA, cardiac valve damage, surgical intervention, medical condition (HT, CHD, T2D, AF at enrolment, and stroke), blood biochemical index (WBC, HGB, PLT, FBG, ALT, AST, Cr, ASO, RF, ESR, BNP, TRIG, TC, LDL-C, HDL-C, serum sodium, and serum potassium), and echocardiography (LAD, LVD, RVD, RAD, and LVEF).*

b *Model 2: It was the same as model 1 and also included combined medication (antiplatelet drugs, warfarin, digoxin, nitrates, RSIs, BBs, MRAs, CCBs, and statins).*

c *Model 3: It was the same as model 2 and also included SDC.*

*The bold values mean P value < 0.05*.

## Discussion

### Association of DGX Application Methods With All-Cause Death, CVD and HF Readmission

Studies or *post-hoc* analyses showed that DGX use was associated with increased adverse cardiovascular events (e.g., all-cause death, CVD, and HF readmission) in HF patients (6, 7, 14, 17). When talking about possible explanations for this harmful effect, it is often attributed to its narrow effective window (18) that can easily cause drug poisoning (19). However, the influence of DGX application approaches on the prognosis of RHD patients with HF remains unclear. After reviewing the data on DGX application approaches from the earliest DIG study to the latest REMEDY study, all studies were designed to use the strategy of cDGX during its 2- to 10-year follow-up regardless of whether the congestion status of HF patients was eliminated or not, which tries to continuously improve heart function and obtain a better survival prognosis. The results of these studies found that cDGX was correlated with increased risks of all-cause death [e.g., by (21–72)%] (6, 7, 14, 17, 20), CVD [e.g., by (25–32)%] (21), and HF readmission [e.g., by (21–41)%] (22) in HF patients. In this real-world, retrospective study, our findings showed that cDGX was indeed related to increased risk of all-cause death (by 84%), CVD (by 1.23-fold), and HF readmission (by 53–61%) compared to iDGX, partially consistent with the recent study by Maciej Tysarowski et al. reporting that DGX use was associated with increased mortality in patients with AF and without concomitant HF *(https://doi.org/10.1161/circ.142.suppl_3.16085)*. Conversely, this suggested that iDGX was associated with better prognosis of RHD patients with HF, which may be the optimal therapy strategy of DGX for those patients.

Interestingly, studies showed that the mortality of patients with DGX was elevated with the increase of SDC (especially ≥ 1.0 ng/ml), and lower SDC (0.5–0.9 ng/ml) may be good for improving the overall risk of death and hospitalization. Under conditions of the two DGX use approaches, however, the effect of different SDC on all-cause mortality and CVD is still unclear. In this study, four issues were worth paying attention to as follows: Firstly, RHD patients with DGX treatment at SDC ≥ 1.0 ng/ml had relatively high risk of all-cause death while they had relatively low risk of CVD, and this phenomenon was observed in both DGX use approaches (Supplementary Table 1). The possible explanation is that the cardiovascular benefits increased with the increases of SDC, but the non-cardiovascular-related deaths increased more obviously, which may be attributed to the poisoning effect at high SDC. Secondly, RHD patients receiving iDGX above the corresponding SDC cutoff value of 0.6 to 1.0 ng/ml had relatively high risk of CVD compared to their counterparts receiving cDGX, especially at SDC ≥ 0.6 ng/ml (Supplementary Table 1). The difference between the two DGX application approaches (iDGX vs. cDGX) was whether or not to receive DGX treatment among RHD patients with HF during their compensated HF period. In other words, those subjects in the iDGX group were re-treated with DGX when congestion occurred again during the follow-up, which meant that they were the “new” users of DGX in one sense. Indeed, a retrospective analyses based on the Apixaban for Reduction in Stroke and Other Thromboembolic Events in Atrial Fibrillation (ARISTOTLE) study reported that new DGX users had significantly higher risk of total mortality (increased by 78%), which was also related to higher sudden cardiac death risk (increased by 3-fold) owing to malignant arrhythmia (23). Those results suggested that iDGX was associated with better prognosis compared to cDGX, but it is necessary to closely monitor the SDC when DGX needs to be reused in HF patients when their cardiac function turns point from compensatory to decompensated stage. Thirdly, the optimal SDC for monitoring of the benefit–risk balance of a treatment in both DGX use approaches was <0.8 ng/ml, because it implies a relatively high cardiovascular benefit and a relatively low risk of all-cause death, which may reflect the interplay of neurohormonal and inotropic effects (18). The finding was consistent with a previous report (24). Finally, the all-cause death risk in male RHD patients with cDGX above the corresponding SDC cutoff value (0.6, 0.8, and 1.0 ng/ml) was approximately increased by 40% compared to that in their female counterparts while CVD risk was at least 90% (Supplementary Table 2). A similar phenomenon was also seen in a previous study (25), but was inconsistent with another study that reported that DGX therapy was related to elevated all-cause death in women (26). The sex-related differences in the effect of DGX among HF patients need further research.

### Association of DGX Application Methods With the Risks of New-Onset AF and Stroke

Many studies have shown that DGX increases predisposition to AF. In AF patients after electrical cardioversion, recurrence risk of AF in those patients receiving DGX increased by 2- to 3-fold, and the duration of AF lasted longer (27). In this study, cDGX was related to elevated risk of new-onset AF (by 1.06-fold), consistent with the above-mentioned study. DGX promotes the increase of calcium load in atrial cells and then increases the “trigger” activity, which is the initiating factor for the electrical remodeling of AF. DGX also promotes fibrosis and inflammation of cardiomyocytes and worsens cardiac structural remodeling, generating a vicious spiral (28).

Evidence confirmed that chronic low-grade inflammation status plays a central role of rheumatism-induced atrial structural and electrical remodeling, leading to higher risk of cardiovascular events (29). After standard antibiotics and surgical treatment, there is no clinical characteristic of rheumatic activity and evidence of subclinical persistent myocarditis in histomorphology, but chronic subclinical active non-specific inflammation still exists in the myocardium, atrium, and valvular tissue (30). A pilot study by Saikia et al. also confirmed the inflammation present in rheumatic valvular diseased tissue was non-specific interstitial inflammation (31). In patients with “end-stage” RHD, the presence of inflammatory cells and the increased expression of multiple cytokines reflected that the damage of chronic low-grade inflammation may be subclinical and sustained damage (32). Chronic subclinical inflammation of RHD was related to the progression of RHD, showing clinical manifestations with left atrial enlargement (LAE), or even “mega LA” (33). On the other hand, recent studies confirmed that the chronic inflammation status associated with uncontrolled rheumatic diseases may be a pathophysiologic driver in the development of metabolic disorder (34), and insulin resistance (IR) is independently associated with rheumatic disease–related inflammation status and metabolic disorder (35). In this study, there were no significant differences in serum level C-reactive protein (CRP), fasting blood glucose (FBG), total cholesterol (TC), and low-density lipoprotein cholesterol (LDL-C) between the two groups (Table 1), but the average levels of CRP (>10.0 mg/L), FBG (>5.6 mmol/L), TC (>4.2 mmol/L), and LDL-C (>1.8 mmol/L) were higher than normal (Table 2). This suggested that RHD patients suffered a status of chronic low-grade inflammation and metabolic disorder (36). At the mouse model level, high-dose DGX promoted inflammation status and then caused myocardial necrosis (37). At the clinical level, a *post-hoc* analysis of the DIG trial found that the substantial risk of DGX toxicity had nothing to do with metabolic status among HF patients (38). The Randomized Evaluation of Long-Term Anticoagulation Therapy (RE-LY) study showed that CRP was a vital risk factor for mortality and vascular accidents in patients with AF (39). These findings suggested that DGX-related AF may be mainly owing to DGX-induced persistent chronic low-grade inflammation status.

Recent studies showed that the mechanism of occurrence and development of AF has far exceeded the influence of atrial-related remodeling but is also related to the adverse effects of coronary microvascular dysfunction (MVD). One of the important mechanisms on coronary microvascular dysfunction (CMD) mainly involves the abnormal production and release of nitric oxide (NO), which is mainly synthesized by endothelial nitric oxide synthase (eNOS) in the cardiovascular system. Under the circumstances of RHD, there is a potential association among DGX use, CMD, and arrhythmia based on NO as follows: Firstly, RHD is an autoimmune disease, with inflammatory factors upregulated, resulting in the inhibition of eNOS activity and then contributing to the reduction of NO bioavailability, which affected the function of coronary microvascular tissue (40). Secondly, Medow et al. (41) found that NO-mediated microvasodilation dysfunction was related to arrhythmia (e.g., postural tachycardia syndrome). Thirdly, DGX use induced AF in eNOS-deficient mice (42). Lastly, intracellular magnesium deficiency may not only be related to MVD (43) but also an important risk factor for AF (44). cDGX may continuously inhibit the Na^+^ -K^+^-ATPase pump, deplete intracellular magnesium ions (45), further lead to CMD, and eventually induce AF.

Theoretically, valvular AF (especially caused by RHD) is associated with increased risk of stroke, even cerebrovascular death. In this study, the two DGX use approaches were not associated with the risks of new-onset stroke (Supplementary Table 3) and cerebrovascular death (Supplementary Table 4). Possible explanations are as follows: Firstly, the main cause of cerebrovascular accident in RHD is cardiogenic embolism caused by LAE. Secondly, LAE is an independent risk factor for thrombosis (46). Thirdly, due to the existence of chronic low-grade inflammation of RHD (47), left atrial remodeling is continuously progressing. However, both iDGX and cDGX had no effect reversing left atrial remodeling and even promoted remodeling. The indifferent serum CRP levels between the two DGX use approaches may explain this at least partially.

### Limitations

This research has produced some relief, but it still has some shortcomings. The main limitation is that this present study was only a retrospective investigation, and participants did not randomly receive DGX application strategies, which may lead to bias (e.g., similar to “per-protocol” analyses present in clinical trials). The neurohumoral condition in RHD patients with iDGX may be better than those with cDGX, which may be linked to better outcomes. This study can only carry out correlation analysis and cannot confirm that DGX application was the reason for increasing poor prognosis. The PSM was performed to eliminate potential bias, but it may also be possible to amplify hidden confounding variables. In addition, because there is still no consensus about the optimal therapy strategy for RHD patients with HF, the evidence-based drugs for HF treatment [e.g., RSIs, beta-receptor blockers (BBs), and MRA] in this retrospective study were not adequate. Hence, the results must be interpreted carefully.

## Conclusion

cDGX was correlated with elevated risk of all-cause death, CVD, medium-/long-term HF readmission, and new-onset AF in RHD patients with HF. In contrast, DGX de-escalation approaches (e.g., iDGX) were related to a better prognosis in RHD patients with HF, suggesting that DGX should be adjusted with a de-escalation therapy as soon as possible after the congestion state of RHD patients with HF is relieved and the clinical condition is stable. It is uncertain whether this relationship observed is due to unnecessary continuous treatment with DGX or simply reflects the inevitable link between more severe HF and more frequent DGX treatment. These substitute hypotheses could be studied in further large well-designed RCTs.

## Data Availability Statement

The raw data supporting the conclusions of this article will be made available by the authors, without undue reservation.

## Ethics Statement

The study received ethics approval from the Institutional Review Board of Guangzhou First People's Hospital, South China University of Technology (K-2018-136-1). Written informed consent for participation was not required for this study in accordance with the national legislation and the institutional requirements.

## Author Contributions

CL: literature search, study format, writing protocol, collecting data, processing data, data interpretation, analyzing data, and writing manuscript. TG and QZ: processing data, data interpretation, and analyzing data. JP, SZ, YL, and DeW: recruiting patients, following up patients, and collecting data. DaW: echocardiography testing. All authors read and approved the final manuscript.

## Funding

This study was funded by the National Natural Science Foundation of China (81100235), the Guangdong Natural Science Foundation of China (S2011040004458), the Guangdong Science and Technology Planning Project of China (2014A020212372), and the Guangzhou Science and Technology Project of China (2012J4100035 and 201804010214).

## Conflict of Interest

The authors declare that the research was conducted in the absence of any commercial or financial relationships that could be construed as a potential conflict of interest. The reviewer RZ declared a shared affiliation, with no collaboration, with the authors to the handling editor at the time of the review.

## Publisher's Note

All claims expressed in this article are solely those of the authors and do not necessarily represent those of their affiliated organizations, or those of the publisher, the editors and the reviewers. Any product that may be evaluated in this article, or claim that may be made by its manufacturer, is not guaranteed or endorsed by the publisher.

## Glossary

AF, atrial fibrillation; ALT, alanine aminotransferase; AR, aortic regurgitation; AS, aortic stenosis; ASO, antistreptolysin O; AST, aspartate aminotransferase; AV, aortic valve; AVR, aortic valve replacement; BBs, beta-receptor blockers; BNP, B-type natriuretic peptide; cDGX, intermittent use of DGX; CHD, coronary heart disease; CI, confidence interval; CCBs, calcium channel blockers; Cr, creatinine; CRP, C-reactive protein; CVD, cardiovascular death; DBP, diastolic blood pressure; DGX, digoxin; eNOS, endothelial nitric oxide synthase; ESR, erythrocyte sedimentation rate; FBG, fasting blood glucose; HDL-C, high-density lipoprotein cholesterol; HF, heart failure; HGB, hemoglobin; HR, hazard ratio; Hr, heart rate; HT, hypertension; LAD, left atrial end-systolic diameter; iDGX, intermittent use of DGX; IR, insulin resistance; LDL-C, low-density lipoprotein cholesterol; LVEF, left ventricular ejection fraction; LVD, left ventricular end-diastolic diameter; MRA, mineralocorticoid receptor antagonist; MR, mitral regurgitation; MS, mitral stenosis; MV, mitral valve; MVD, microvascular dysfunction; NYHA, New York Heart Association; NO, nitric oxide; OR, odds ratio; PAH, pulmonary arterial hypertension; PLT, platelet count; PSM, propensity score matching; RAD, right atrial end-systolic diameter; RHD, rheumatic heart disease; RF, rheumatoid factor; RSIs, renin–angiotensin system inhibitors; RVD, right ventricular end-diastolic diameter; SBP, systolic blood pressure; TC, total cholesterol; T2D, type 2 diabetes mellitus; TR, tricuspid regurgitation; TV, tricuspid valve; TRIG, triglyceridemia; VAF, valvular atrial fibrillation; WBC, white blood cell count.
