# Supplementary material for: Association of Digoxin Application Approaches With Long-Term Clinical Outcomes in Rheumatic Heart Disease Patients With Heart Failure: A Retrospective Study
Source: Front Cardiovasc Med. 2021 Sep 20;8:711203. doi: 10.3389/fcvm.2021.711203 (PMC8488133; doi:10.3389/fcvm.2021.711203)
Supplement: Supplementary file 1 [file Data_Sheet_1.PDF]

**Supplementary Material**

## Methods

### Primary and secondary outcomes

All-cause death, ardiovascular death (CVD) and cerebrovascular death were the primary outcomes. The all-cause death defined as death due to any causes. The CVD was defined as death due to any cardio-/cerebro-vascular diseases, such as CHD, HF, AF, stroke and or other cardio-/cerebro-vascular causes. The cerebrovascular death was defined as was defined as death due to cerebral infarction or cerebral hemorrhage. HF readmission, new-onset AF and new-onset stroke were the secondary outcomes. After first discharge for HF, HF readmission was defined as hospital re-hospitalization with HF treatment. The new-onset AF was defined as AF that first occurred during follow-up period. The new-onset stroke was defined as the presence of a focal/global neurological event with symptoms and signs lasting > 24 h including hemorrhagic stroke, ischemic stroke, cardiogenic stroke or transient ischemic attack, and determined by magnetic resonance image and/or computed tomography scanning of the brain.

### Statistical analysis

All statistical analyses were performed using SPSS version 24 (SPSS, Chicago, IL). Categorical and continuous variables were showed as numbers (percentages) and mean ± standard deviation (SD), respectively. Significant differences for categorical variables and continuous variables were determined by $\chi^2$ test and independent-sample t-test, respectively. The Cox proportional hazards regression model was carried out to access the association [hazard ratio (HR) and 95% confidence interval (CI)] of different DGX use methods with all-cause death, CVD and cerebrovascular death, adjusting by baseline characteristics. Binary logistic regression model was carried out to evaluate the association [Odds ratio (OR) and 95%

CI] of different DGX use methods with 1-year, 3-year and 5-year HF readmission, new-onset AF and new-onset stroke. A *P* value of 0.05 or less was statistically significant (two-tailed).

**Results**

**Association of SDC with all-cause death and CVD in RHD patients**

To access the effect of different SDC on all-cause mortality and CVD under the two DGX application approaches, we further performed multivariate Cox regression analyses based on the SDC (0.6 to 1.0 ng/mL) by cutoff value in every 0.1 ng/mL after PSM, as shown in Table S1. RHD patients with DGX at SDC ≥ 0.6 ng/mL showed increased all-cause death risk (adjusted HR=1.87, 95% CI: 1.17-2.98, *P*=0.009) compared to those at SDC < 0.6 ng/mL. The changes of the harmful effect were not obvious at SDC cutoff value of 0.6 to 0.9 ng/mL with the exception of 1.0 ng/mL (adjusted HR=2.13, 95% CI: 1.11-4.07, *P*=0.023). In particular, the all-cause mortality risk (adjusted HR=3.58, 95% CI: 1.24-10.30, *P*=0.018) among participants at SDC ≥ 1.0 ng/mL in iDGX subgroup was obvious increased compared to their counterparts, and the effect was much worse in cDGX subgroup (adjusted HR=4.58, 95% CI: 1.33-15.78, *P*=0.016). There was no adverse effect on all-cause mortality at SDC cutoff value of 0.6 and 0.7 ng/mL in iDGX subgroup.

On the other hand, RHD patients with DGX at SDC ≥ 0.6 ng/mL also showed increased CVD risk (adjusted HR=2.10, 95% CI: 1.20-3.69, *P*=0.009) compared to those at SDC < 0.6 ng/mL. The elevated intensity in CVD risk gradually decreased with the increase in SDC cutoff value of 0.6 to 1.0 ng/mL, and there was a relatively low increased CVD risk at SDC cutoff value of 1.0 ng/mL (adjusted HR=1.65, 95% CI: 1.08-2.51, *P*=0.020). Interestingly, the increases of CVD risk among participants above corresponding SDC cutoff value in iDGX subgroup were obviously higher than their counterparts in cDGX subgroup, especially at SDC cutoff value of 0.6 ng/mL as follows: iDGX (adjusted HR=5.12, 95% CI: 1.13-23.14, *P*=0.034) vs. cDGX (adjusted HR=2.06, 95% CI: 1.06-4.03, *P*=0.033).

**Association of DGX application methods with all-cause death and CVD in RHD patients base on gender**

As shown in Table S2, cDGX was associated with increased risks of all-cause death (adjusted HR=1.88, 95% CI: 1.32-2.68, $P<0.001$) and CVD (adjusted HR=2.99, 95% CI: 1.82-4.91, $P<0.001$) among RHD patients with DGX treatment at SDC ≥ 0.6 ng/mL compared to their counterparts with iDGX. The changes of the harmful effect among RHD patients with cDGX were elevated with the increase of SDC. In RHD patients with cDGX above corresponding SDC cutoff value of 0.6, 0.8 and 1.0 ng/mL, cDGX related harmful effect on all-cause mortality and CVD risk in male RHD patients were significant worse than that in female counterparts, especially at SDC cutoff value of 1.0 ng/mL as follows: all-cause mortality (adjusted HR=2.99, 95% CI: 1.75-5.11, $P<0.001$) and CVD (adjusted HR=6.32, 95% CI: 1.08-36.88, $P=0.041$).

**Association of different DGX application methods with new-onset stroke in RHD patients**

As shown in Table S3, the two DGX application approaches had nothing to do with the risk of new-onset stroke before PSM (OR=0.62, 95% CI: 0.36-1.05, $P=0.077$) and after PSM (OR=0.71, 95% CI: 0.42-1.22, $P=0.213$).

**Association of different DGX application methods with cerebrovascular death in RHD patients**

As shown in Table S4, the two DGX application approaches had nothing to do with the risk of cerebrovascular death before PSM (HR=0.73, 95% CI: 0.25-2.11, $P=0.562$) and after PSM (HR=0.72, 95% CI: 0.24-2.14, $P=0.553$).

## Supplementary Table legends

Table S1. Association of SDC with all-cause death and CVD in RHD patients.

Table S2. Association of DGX application methods with all-cause death and CVD in RHD patients base on gender.

Table S3. Association of different DGX application methods with new-onset stroke in RHD patients.

Table S4. Association of different DGX application methods with cerebrovascular death in RHD patients.

**Table S1. Association of SDC with all-cause death and CVD in RHD patients[a].**

| Outcomes | SDC (ng/mL) | Total | | | iDGX | | | cDGX | | |
|---|---|---|---|---|---|---|---|---|---|---|
| | | Events (N/%) | 95% CI | *P* value | Events (N/%) | 95% CI | *P* value | Events (N/%) | 95% CI | *P* value |
| **All-cause death** | <0.6(Ref.)/≥ 0.6 | 34(20.9)/162(33.8) | 1.87(1.17-2.98) | 0.009 | 11(11.6)/55(24.3) | 2.03(0.95-4.33) | 0.068 | 23(33.8)/107(42.3) | 2.29(1.06-4.96) | 0.036 |
| | <0.7(Ref.)/≥ 0.7 | 52(25.1)/144(33.1) | 1.65(1.08-2.51) | 0.019 | 17(14.7)/49(23.9) | 1.74(0.89-3.38) | 0.105 | 35(38.5)/95(41.3) | 2.02(1.06-3.85) | 0.033 |
| | <0.8(Ref.)/≥ 0.8 | 69(27.0)/127(32.9) | 1.70(1.15-2.52) | 0.008 | 24(16.8)/42(23.6) | 2.12(1.12-4.02) | 0.021 | 45(39.8)/85(40.9) | 2.09(1.13-3.86) | 0.018 |
| | <0.9(Ref.)/≥ 0.9 | 81(27.6)/115(33.0) | 1.71(1.17-2.50) | 0.005 | 26(16.4)/40(24.7) | 2.45(1.31-4.59) | 0.005 | 55(41.0)/75(40.1) | 2.07(1.14-3.77) | 0.017 |
| | <1.0(Ref.)/≥ 1.0 | 97(29.0)/99(32.1) | 2.13(1.11-4.07) | 0.023 | 33(18.2)/33(23.6) | 3.58(1.24-10.30) | 0.018 | 64(41.8)/66(39.3) | 4.58(1.33-15.78) | 0.016 |
| CVD | <0.6(Ref.)/≥ 0.6 | 16(9.8)/102(21.3) | 2.10(1.20-3.69) | 0.009 | 2(2.1)/29(12.8) | 5.12(1.13-23.14) | 0.034 | 14(20.6)/73(28.9) | 2.06(1.06-4.03) | 0.033 |
| | <0.7(Ref.)/≥ 0.7 | 25(12.1)/93(21.4) | 1.86(1.15-3.01) | 0.011 | 6(5.2)/25(12.2) | 3.19(1.10-9.23) | 0.032 | 19(20.9)/68(29.6) | 2.03(1.12-3.68) | 0.020 |
| | <0.8(Ref.)/≥ 0.8 | 33(12.9)/85(22.0) | 1.87(1.20-2.91) | 0.006 | 9(6.3)/22(12.4) | 2.91(1.10-7.66) | 0.031 | 24(21.2)/63(30.3) | 1.95(1.12-3.40) | 0.018 |
| | <0.9(Ref.)/≥ 0.9 | 42(14.3)/76(21.8) | 1.71(1.11-2.64) | 0.016 | 10(6.3)/21(13.0) | 3.27(1.20-8.88) | 0.020 | 32(23.9)/55(29.4) | 1.79(1.02-3.14) | 0.044 |
| | <1.0(Ref.)/≥ 1.0 | 54(16.2)/64(20.8) | 1.65(1.08-2.51) | 0.020 | 14(7.7)/17(12.1) | 2.66(1.03-6.86) | 0.043 | 40(26.1)/47(28.0) | 1.78(1.03-3.08) | 0.040 |

[a]Model 3a: adjusting for baseline adjustment covariates, including age, gender, smoking, drinking, disease duration, NYHA, cardiac valve damage, surgical intervention, medical condition (HT, CHD, T2D, AF at enrolment, new-onset AF, and stroke), combined medication (antiplatelet drugs, warfarin, digoxin, nitrates, RSIs, BBs, MRAs, CCBs, and statins), blood biochemical index (WBC, HGB, PLT, FBG, ALT, AST, Cr, ASO, RF, ESR, BNP,TRIG, TC, LDL-C, HDL-C, serum sodium, and serum potassium), echocardiography (LAD, LVD, RVD, RAD, and LVEF) and SDC, but stratified by SDC of 0.6 to 1.0 ng/mL by cutoff value in every 0.1 ng/mL, respectively.

**Table S2. Association of DGX application methods with all-cause death and CVD in RHD patients base on gender[b].**

| Outcomes | SDC (ng/mL) | Group | Total | | | Female | | | Male | | |
|---|---|---|---|---|---|---|---|---|---|---|---|
| | | | Events (N/%) | 95% CI | *P* value | Events (N/%) | 95% CI | *P* value | Events (N/%) | 95% CI | *P* value |
| **All-cause death** | ≥ 0.6 | iDGX | 55(24.3) | Ref. | | 36(22.2) | Ref. | | 19(29.7) | | |
| | | cDGX | 107(42.3) | 1.88(1.32-2.68) | <0.001 | 68(37.8) | 1.80(1.15-2.81) | 0.010 | 39(54.3) | 2.55(1.21-5.36) | 0.014 |
| | ≥ 0.8 | iDGX | 42(23.6) | Ref. | | 30(22.7) | Ref. | | 12(26.1) | | |
| | | cDGX | 85(40.9) | 2.02(1.34-3.05) | 0.001 | 56(37.1) | 1.91(1.12-3.26) | 0.017 | 29(50.9) | 2.78(1.15-6.73) | 0.024 |
| | ≥ 1.0 | iDGX | 33(23.6) | Ref. | | 22(21.6) | Ref. | | 11(28.9) | | |
| | | cDGX | 66(39.3) | 2.30(1.69-3.13) | <0.001 | 41(34.2) | 2.08(1.40-3.08) | <0.001 | 25(52.1) | 2.99(1.75-5.11) | <0.001 |
| CVD | ≥ 0.6 | iDGX | 29(12.8) | Ref. | | 20(12.3) | Ref. | | 9(14.1) | | |
| | | cDGX | 73(28.9) | 2.99(1.82-4.91) | <0.001 | 45(25.0) | 2.46(1.29-4.67) | 0.002 | 28(38.4) | 4.90(1.52-15.77) | 0.008 |
| | ≥ 0.8 | iDGX | 22(12.4) | Ref. | | 16(12.1) | Ref. | | 6(13.0) | | |
| | | cDGX | 63(30.3) | 3.05(1.80-5.17) | <0.001 | 40(26.5) | 2.69(1.39-5.19) | 0.003 | 23(40.4) | 5.09(1.47-17.59) | 0.010 |
| | ≥ 1.0 | iDGX | 17(12.1) | Ref. | | 11(10.8) | Ref. | | 6(15.8) | | |
| | | cDGX | 47(28.0) | 3.27(1.78-6.02) | <0.001 | 28(23.3) | 3.19(1.40-7.24) | 0.006 | 19(39.6) | 6.32(1.08-36.88) | 0.041 |

[b]Model 3b: adjusting for baseline adjustment covariates, including age, smoking, drinking, disease duration, NYHA, cardiac valve damage, surgical intervention, medical condition (HT, CHD, T2D, AF at enrolment, new-onset AF, and stroke), combined medication (antiplatelet drugs, warfarin, digoxin, nitrates, RSIs, BBs, MRAs, CCBs, and statins), blood biochemical index (WBC, HGB, PLT, FBG, ALT, AST, Cr, ASO, RF, ESR, BNP,TRIG, TC, LDL-C, HDL-C, serum sodium, and serum potassium), echocardiography (LAD, LVD, RVD, RAD, and LVEF) and SDC, but stratified by gender.

**Table S3. Association of different DGX application methods with new-onset stroke in RHD patients.**

| New-onset stroke | | Before PSM | | | After PSM | | |
|---|---|---|---|---|---|---|---|
| | | Events (N/%) | OR (95% CI) | *P* value | Events (N/%) | OR (95% CI) | *P* value |
| **Unadjusted** | **iDGX** | 49(15.9) | Ref. | | 46(16.3) | Ref. | |
| | **cDGX** | 44(12.8) | 0.77(0.50-1.20) | 0.250 | 42(14.8) | 0.89(0.57-1.41) | 0.630 |
| **Model 1[a]** | **iDGX** | 49(15.9) | Ref. | | 46(16.3) | Ref. | |
| | **cDGX** | 44(12.8) | 0.70(0.42-1.16) | 0.163 | 42(14.8) | 0.78(0.47-1.30) | 0.345 |
| **Model 2[b]** | **iDGX** | 49(15.9) | Ref. | | 46(16.3) | Ref. | |
| | **cDGX** | 44(12.8) | 0.63(0.37-1.07) | 0.089 | 42(14.8) | 0.72(0.43-1.23) | 0.230 |
| **Model 3[c]** | **iDGX** | 49(15.9) | Ref. | | 46(16.3) | Ref. | |
| | **cDGX** | 44(12.8) | 0.62(0.36-1.05) | 0.077 | 42(14.8) | 0.71(0.42-1.22) | 0.213 |

[a]Model 1: adjusting for baseline adjustment covariates, including age, gender, smoking, drinking, disease duration, NYHA, cardiac valve damage, surgical intervention, medical condition (HT, CHD, T2D, AF at enrolment and stroke at enrolment), blood biochemical index (WBC, HGB, PLT, FBG, ALT, AST, Cr, ASO, RF, ESR, BNP,TRIG, TC, LDL-C, HDL-C, serum sodium, and serum potassium) and echocardiography (LAD, LVD, RVD, RAD, and LVEF).
[b]Model 2: It was same as Model 1, and also including new-onset AF and combined medication (antiplatelet drugs, warfarin, digoxin, nitrates, RSIs, BBs, MRAs, CCBs, and statins).
[c]Model 3: It was same as Model 2, and also including SDC.

**Table S4. Association of different DGX application methods with cerebrovascular death in RHD patients.**

| Cerebrovascular death | | Before PSM | | | After PSM | | |
|---|---|---|---|---|---|---|---|
| | | Events (N/%) | HR (95% CI) | *P* value | Events (N/%) | HR (95% CI) | *P* value |
| **Unadjusted** | iDGX | 14 (4.0) | Ref. | | 13(4.0) | Ref. | |
| | cDGX | 13(3.4) | 1.06(0.50-2.26) | 0.880 | 12(3.7) | 1.17(0.53-2.56) | 0.702 |
| **Model 1**[a] | iDGX | 14 (4.0) | Ref. | | 13(4.0) | Ref. | |
| | cDGX | 13(3.4) | 0.86(0.34-2.19) | 0.756 | 12(3.7) | 0.91(0.35-2.41) | 0.851 |
| **Model 2**[b] | iDGX | 14 (4.0) | Ref. | | 13(4.0) | Ref. | |
| | cDGX | 13(3.4) | 0.76(0.27-2.11) | 0.597 | 12(3.7) | 0.74(0.26-2.16) | 0.586 |
| **Model 3**[c] | iDGX | 14 (4.0) | Ref. | | 13(4.0) | Ref. | |
| | cDGX | 13(3.4) | 0.73(0.25-2.11) | 0.562 | 12(3.7) | 0.72(0.24-2.14) | 0.553 |

[a]Model 1: adjusting for baseline adjustment covariates, including age, gender, smoking, drinking, disease duration, NYHA, cardiac valve damage, surgical intervention, medical condition (HT, CHD, T2D, AF at enrolment, and stroke at enrolment), blood biochemical index (WBC, HGB, PLT, FBG, ALT, AST, Cr, ASO, RF, ESR, BNP,TRIG, TC, LDL-C, HDL-C, serum sodium, and serum potassium) and echocardiography (LAD, LVD, RVD, RAD, and LVEF).
[b]Model 2: It was same as Model 1, and also including new-onset AF, new-onset stroke and combined medication (antiplatelet drugs, warfarin, digoxin, nitrates, RSIs, BBs, MRAs, CCBs, and statins).
[c]Model 3: It was same as Model 2, and also including SDC.
